# Novel utilization of deep brain stimulation in the pedunculopontine nucleus with globus pallidus internus for treatment of childhood-onset dystonia

**DOI:** 10.3389/fnhum.2023.1270430

**Published:** 2023-10-19

**Authors:** Jennifer A. MacLean, Jaya Nataraj, Jordan Davies, Aleksandra Zakharova, Joshua Kurtz, Mark A. Liker, Joffre Olaya, Terence D. Sanger

**Affiliations:** ^1^Department of Neurology, Children’s Hospital of Orange County, Orange, CA, United States; ^2^Research Institute, Children’s Hospital of Orange County, Orange, CA, United States; ^3^Samueli School of Engineering, University of California, Irvine, Irvine, CA, United States; ^4^Division of Neurosurgery, Children’s Hospital of Orange County, Orange, CA, United States; ^5^Department of Neurological Surgery, School of Medicine, University of California, Irvine, Irvine, CA, United States; ^6^Unit of Pediatric Neurology, Faculty of Medicine Universidad del Desarrollo, Clínica Alemana de Santiago, Santiago, Chile; ^7^School of Medicine, University of California, Irvine, Irvine, CA, United States; ^8^Department of Neurological Surgery, Keck School of Medicine, University of Southern California, Los Angeles, CA, United States; ^9^Department of Pediatrics, School of Medicine, University of California, Irvine, Irvine, CA, United States

**Keywords:** dystonia, pediatrics, pedunculopontine nucleus, deep brain stimulation, orofacial dyskinesia, stereotaxy

## Abstract

**Introduction:**

Deep brain stimulation (DBS) is a well-documented therapy for dystonia utilized in many adult and pediatric movement disorders. Pedunculopontine nucleus (PPN) has been investigated as a DBS target primarily in adult patients with dystonia or dyskinesias from Parkinson’s disease, showing improvement in postural instability and gait dysfunction. Due to the difficulty in targeting PPN using standard techniques, it is not commonly chosen as a target for adult or pediatric pathology. There is no current literature describing the targeting of PPN in DBS for childhood-onset dystonia.

**Methods:**

Two pediatric and one young adult patient with childhood-onset dystonia who underwent DBS implantation at our institution were identified. Patient 1 has Mitochondrial Enoyl CoA Reductase Protein-Associated Neurodegeneration (MEPAN) syndrome. Patient 2 has Glutaric Aciduria Type 1 (GA1). Patient 3 has atypical pantothenate kinase-associated neurodegeneration (PKAN). PPN was identified as a potential target for these patients due to axial or orofacial dystonia. Pre- and post-operative videos taken as part of routine clinical assessments were evaluated and scored on the Burke-Fahn-Marsden Dystonia Rating Scale (BFMDRS) and Barry-Albright Dystonia Scale (BADS). All patients had permanent electrodes placed bilaterally in PPN and globus pallidus internus (GPi). A Likert scale on quality of life was also obtained from the patient/parents as applicable.

**Results:**

Significant programming was necessary over the first 3–12 months to optimize patients’ response to stimulation. All patients experienced at least a 34% improvement in the BFMDRS score. Patients 2 and 3 also experienced an over 30% improvement in BADS score. All patients/parents appreciated improvement in quality of life postoperatively.

**Discussion:**

Deep brain stimulation in PPN was safely and successfully used in two pediatric patients and one young adult patient with childhood-onset dystonia. These patients showed clinically significant improvements in BFMDRS scoring post operatively. This represents the first reported DBS targeting of PPN in pediatric patients, and suggests that PPN is a possible target for pediatric-onset dystonia with axial and orofacial symptoms that may be refractory to traditional pallidal stimulation alone.

## 1. Introduction

Deep brain stimulation (DBS) is a surgical technique commonly used to treat medically refractory dystonia in children and adults. Initially approved for utilization in treatment of Parkinson’s disease (PD), DBS was approved for treatment of dystonia in 2003 (U.S. Food and Drug Administration, Center for Devices and Radiological Health, 2003). Dystonia can be classified as primary, occurring without other brain abnormalities, or secondary, when it is associated with central nervous system injury due to a wide variety of potential causes. The treatment efficacy of DBS is well-established for certain genetic pediatric-onset primary dystonia, and current literature shows selection of the globus pallidus internus (GPi) as the primary target for stimulation generally results in some degree of improvement in motor symptoms (Kupsch et al., 2006; Ostrem and Starr, 2008). However, treatment of other pediatric-onset dystonias with DBS is rarely as straightforward, since the multitude of possible origins can result in several different clinical presentations of dystonia. The identification of optimal stimulation targets for these dystonic conditions remains an open question in the field since the reported outcomes on use of GPi DBS are limited in scope and consistency (Ostrem and Starr, 2008).

Several additional possibilities for DBS targets have been identified and are now commonly used, including subthalamic nucleus (STN), ventrolateral thalamus (VL), ventral intermediate nucleus of the thalamus (VIM), and ventralis oralis anterior (Voa) and posterior (Vop) nuclei of the thalamus (Ostrem and Starr, 2008; Vitek et al., 2011). Use of these targets in treatment of dystonia has yielded varied results, suggesting that the specified thalamic and basal ganglia targets are suitable for consideration, but that optimal targets for stimulation may be patient specific (Krack and Vercueil, 2001; Katsakiori et al., 2009). It is also likely that optimal targets may have to be identified based on the specific distribution and symptomatology of dystonia. One key example is in patients presenting with orofacial and axial symptoms. Although stimulation in standard pallidal targets was able to elicit some improvement in orofacial deficits in subjects with secondary dystonia, the results were not comparable to the level of benefit achieved in treatment of primary dystonia (Castelnau et al., 2005). A similarly dissatisfactory result is seen in patients with PD who display axial motor deficits, which are poorly responsive to the commonly utilized targets of STN and GPi (Thevathasan et al., 2018). The lack of response of these deficits to standard targets has motivated the search for novel DBS targets.

Deep brain stimulation in the pedunculopontine nucleus (PPN) was first identified as an experimental therapy to treat axial motor symptoms in PD, since PPN is a major component of the mesencephalic locomotor region and is thought to play a role in gait and production of movement (Thevathasan et al., 2018). Although initially an exciting prospect, the reported outcomes on PPN DBS for PD patients were largely inconclusive (Thevathasan et al., 2018). Despite this anticlimactic result in the PD population, there is reason to believe that PPN DBS could provide therapeutic benefit in the treatment of other motor disorders, such as secondary dystonia. Although the specific mechanisms of dystonia are unknown, it is often characterized as a network disorder involving the basal ganglia-cerebello-thalamo-cortical circuit (Ostrem and Starr, 2008; Su et al., 2022).

The PPN is a brainstem structure located in the caudal mesencephalic tegmentum, and it displays widespread reciprocal anatomical connections to the cerebral cortex, thalamus, basal ganglia, motor regions of the brainstem, and spinal cord (Alam et al., 2011; Rowe et al., 2016; Nowacki et al., 2019). The PPN is separated into the rostral and caudal sections with the former containing mainly GABAergic neurons, the latter containing mainly glutamatergic neurons, and intermingled cholinergic neurons throughout the entire structure (Nowacki et al., 2019). The PPN is anatomically and functionally relevant to dystonia due to its complex ascending connections with the basal ganglia and cerebellum, which are thought to play a role in selection and coordination of movements (Su et al., 2022). The PPN is also believed to have descending connections to cranial nerve nuclei V, VII, and XII, as well as to effectors in the spinal cord (Su et al., 2022). These descending projections to areas driving tongue, facial, and trunk musculature allow us to identify PPN as an interesting potential target for treatment of orofacial and axial features of dystonia.

The complex and widespread connectivity of PPN suggests that it is also implicated in several non-motor functions such as regulation of the sleep–wake cycle and attentional networks such as the reticular activating system (Garcia-Rill et al., 2015; Nowacki et al., 2019). This raises the concern for possible nonmotor benefits and side effects. Possible non-motor effects of PPN DBS include promotion of rapid eye movement (REM) sleep, related to the enhancement of the acetylcholine releasing subpopulation of neurons within PPN that may affected by specific frequencies of stimulation (Rye, 1997). Increased REM sleep has been observed in the Parkinson’s disease population with PPN stimulation, though without a change in the presence of REM sleep behavior disorder or overall total sleep time, suggesting involvement of multiple pathways (Lim et al., 2009). Additionally, the proximity of PPN to the pontine micturition center suggests that PPN DBS may induce undesirable urinary side effects, previously reported in PD patients (Aviles-Olmos et al., 2011; Thevathasan et al., 2018). Other previously reported adverse effects of PPN DBS include contralateral paresthesia, sensation of pain, oscillopsia, and limb myoclonus (Nowacki et al., 2019).

The vast involvement of the PPN in central nervous system anatomical and functional networks, in conjunction with its relatively small size and the difficulty involved in targeting it using standard neurosurgical techniques suggest that PPN DBS could very well be a double-edged sword (Welter et al., 2015). While it shows promise as an emerging therapy, it is clear that optimal targeting of PPN to increase benefit and diminish side effects will depend heavily on methodological considerations such as electrode size, stimulation voltage and frequency, and the use of stimulation cycling parameters. We report the response of three subjects, two pediatric and one young adult, with dystonia of heterogeneous etiologies receiving combined stimulation in GPi and PPN. To the best of our knowledge, there is no current literature describing the targeting of PPN in DBS for childhood-onset dystonia.

## 2. Materials and methods

### 2.1. Subjects

Two pediatric and one young adult patient with childhood-onset dystonia who underwent DBS implantation at our institution were identified. All three patients were previously diagnosed with dystonia by a pediatric movement disorder specialist (TDS) based on established criteria (Albanese et al., 2013). All had failed standard pharmacotherapy at adequate dosing (Luc and Querubin, 2017) as well as botulinum toxin injections.

Patient 1 is a male with Mitochondrial Enoyl CoA Reductase Protein-Associated Neurodegeneration (MEPAN) syndrome diagnosed by whole genome sequencing. He was 10 years old at the time of DBS placement. His predominant symptoms were axial and appendicular dystonic posturing. He had very limited speech due to severe dysarthria, but was able to utilize an assistive communication device and was performing at grade level. Due to axial posturing he was unable to ambulate with or without support, sit comfortably in his wheelchair, or independently perform many activities of daily living including feeding himself.

Patient 2 is a male with glutaric aciduria type I (GA1) who had an initial metabolic crisis as an infant prior to diagnosis by genetic testing. His predominant symptoms were orofacial dyskinesias and axial posturing that were interfering with his ability to sit comfortably in a wheelchair or initiate sleep. He was 8 years old at the time of implantation. He was appreciated to have significant cognitive delays and limited communication.

Patient 3 is a 23 year old male diagnosed with atypical pantothenate kinase-associated neurodegeneration (PKAN). He was noted to have normal cognition and communicated by speech despite severe dysarthria. His predominant concerns were orofacial and oropharyngeal dystonia interfering with eating and speech. He had multiple episodes of choking requiring the Heimlich maneuver at home. Despite extensive discussions with multiple subspecialists at multiple institutions he refused consideration of a gastronomy tube and instead requested consideration for deep brain stimulation to address the dystonic spasms limiting his oral intake.

Patients or parents of minor patients consented to surgical procedures according to standard hospital consent procedures. They also consented or assented as appropriate to HIPAA authorization for research use of protected healthcare information and IRB-approved consent for videotaping and scale scoring of video recordings.

### 2.2. Surgical procedure

As all three patients had dystonia due to a condition with either known inadequate response to pallidal stimulation alone (GA1, atypical PKAN) or no known response to DBS (MEPAN), it was elected to perform a previously described staged surgical target identification method (Sanger et al., 2018a,b; MacLean et al., 2021, 2023). All subjects were initially implanted with 12 depth electrodes (six bilaterally) in possible targets of pallidum, thalamus, subthalamic nucleus, and PPN. PPN was identified as providing optimal benefit in conjunction with globus pallidus internus (GPi) stimulation on all three patients’ major debilitating dystonic symptoms during a 4–6 day inpatient hospitalization with externalized depth electrodes in which stimulation of various areas was trialed to assess clinical response. PPN was specifically targeted in these patients based on the literature regarding benefits associated with stimulation in PD patients and the lack of response of orofacial and axial dystonia to typical pallidal and thalamic targets. All subjects also concurrently had leads implanted in bilateral GPi based on their response to test stimulation.

Stereotaxy for both depth and permanent electrodes was performed using the ROSA surgical robot with guidance from ONE™ software (Zimmer Biomet, Montpellier, France). Targeting was performed using standard surgical anatomical Schaltenbrand-Wahren atlas locations relative to the AC-PC line. Initially, PPN was targeted based on standard ACPC coordinates (Thevathasan et al., 2018), however targets were subsequently adjusted based on each patient’s individual anatomy on a high-resolution pre-operative MRI. The coordinates of each patient and the standard coordinates are noted in Table 1. Targeting was confirmed by intraoperative fluoroscopy and postoperative CT.

**Table 1:** ACPC coordinates of PPN and GPi electrodes relative to mid-commissural point.

	Right PPN electrode	Left PPN electrode	Right GPi electrode	Left GPi electrode
Standard	*x* = 6.5	*x* = −6.5		
	*y* = −15	*y* = −15		
	*z* = −14	*z* = −14		
Patient 1	*x* = 4.04	*x* = −3.29	*x* = 21.45	*x* = −15.85
	*y* = −17.21	*y* = −16.78	*y* = 5.19	*y* = 7.95
	*z* = −13.04	*z* = −16.74	*z* = −4.90	*z* = −4.01
Patient 2	*x* = 2.08	*x* = −4.64	*x* = 16.33	*x* = −15.31
	*y* = −14.91	*y* = −15.94	*y* = 4.27	*y* = 1.32
	*z* = −12.34	*z* = −15.93	*z* = −1.64	*z* = −1.67
Patient 3	*x* = 5.08	*x* = −5.68	*x* = 11.68	*x* = −13.02
	*y* = −18.67	*y* = −16.12	*y* = 6.22	*y* = 8.33
	*z* = −14.81	*z* = −14.02	*z* = 0.48	*z* = 2.29

Patient 1 was implanted with bilateral Sensight1.5 electrodes (Medtronic Inc., Minneapolis, MN, United States) while patients 2 and 3 were each implanted with bilateral Sensight0.5 electrodes (Medtronic Inc.) in PPN to allow more precise stimulation patterns due to closer spaced stimulation contacts, based on the response of patient 1. All patients were concurrently implanted with two bilateral Sensight1.5 electrodes (Medtronic Inc.) in GPi. Given the utilization of segmented contacts on electrodes (in the two medial contacts of the four contact electrodes), adjustments were also made to ensure segmented contacts were in the targeted region. Trajectories of GPi electrodes were adjusted to allow for both leads in each hemisphere to exit through the same burr hole. Two weeks following placement of permanent electrodes, B34000 Sensight extensions (Medtronic Inc.) were connected to the intracranial electrodes and tunneled subcutaneously to implanted pulse generators (Medtronic Activa RC) placed in the chest. Homologous leads were directed to the same pulse generator (i.e., both PPN electrodes to the right generator).

### 2.3. Scales

All patients were assessed utilizing the Burke-Fahn-Marsden Dystonia Rating Scale (Burke et al., 1985; BFMDRS) and Barry-Albright Dystonia Scale (Barry and Vanswearingen, 1999; BADS) prior to surgery and at least 3 months postoperatively. Assessment was made by video review by a single clinician both preoperatively and postoperatively and then confirmed independently by a second clinician. Agreement from both scores was required for validation. Videos could not be blinded as patients showed visible effects of aging and surgical interventions.

### 2.4. Stimulation parameters

**Figure 1:**
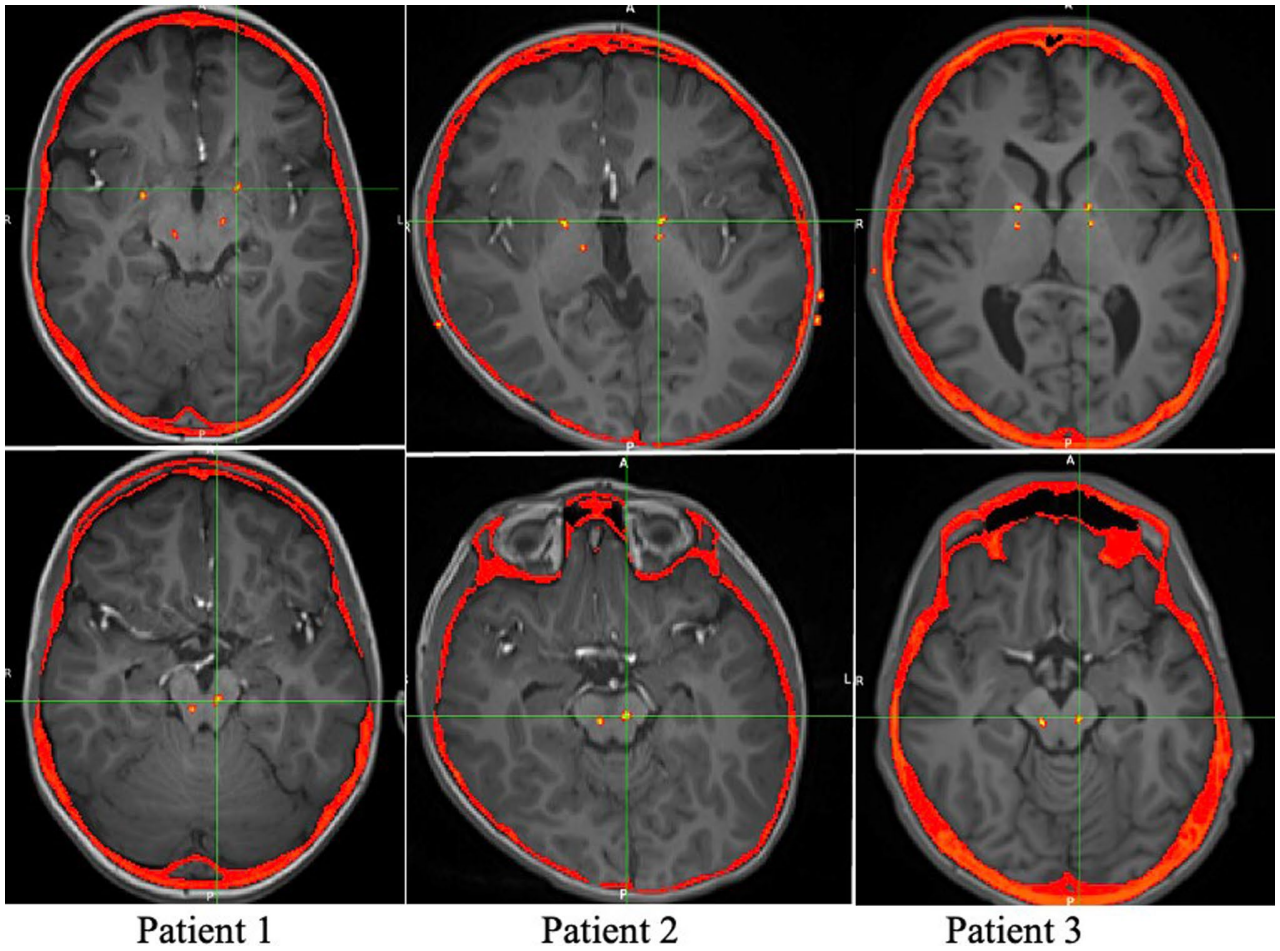
Axial views of the postoperative CT overlaid on the preoperative MRI, showing the locations of the Medtronic leads.

At the time of generator placement low voltage stimulation was initiated on single circumferential monopolar contacts in GPi (1 v) and PPN (0.2 v) bilaterally. Contacts were selected based on location within the targeted structure by merge of the postoperative CT scan with a preoperative high-resolution MRI, shown in Figure 1. The patients were seen at 2 and 4 weeks postoperatively for initial mapping, followed by visits every 1–4 weeks for further programming.

**Table 2:** Stimulation parameters in PPN and GPi at time of post-operative assessment.

	Left PPN	Right PPN	Left GPi	Right GPi
Patient 1 (11 months post-operative)	Cycling with stimulation on for 1 min and off for 5 min	cycling with stimulation on for 1 min and off for 5 min	1b-2a-2c—case+	8-9c-10c—case+
	1c—case +	9c—case +	0.2 v	0.4 v
	0.2 v	0.2 v	90 μs	90 μs
	60 μs	60 μs	185 Hz	250 Hz
	250 Hz	250 Hz		
Patient 2 (3.5 months post-operative)	Cycling with stimulation on for 1 min and off for 5 min	Cycling with stimulation on for 1 min and off for 5 min	2—case+	9—case+
	2a—case+	10a—case+	2.5 v	2.5 v
	0.2 v	0.2 v	90 μs	90 μs
	60 μs	60 μs	190 Hz	190 Hz
	30 Hz	30 Hz		
Patient 3 (3.5 months post-operative)	Cycling with stimulation on for 0.1 s and off for 0.1 s	Cycling with stimulation on for 0.1 s and off for 0.1 s	01—case+	8—case+
	1c-2c—case+	9a—case+	3 v	3 v
	0.4 v	0.4 v	90 μs	60 μs
	50 μs	60 μs	185 Hz	185 Hz
	40 Hz	40 Hz		

#### 2.4.1. Mapping

For mapping of PPN, all stimulation was initially turned off for 15 min. PPN stimulation was then reinitiated unilaterally with each contact including segmented contacts activated individually in monopolar stimulation. Initial stimulation was performed at 0.1 v, 40 Hz, and 60 μs. Benefits and side effects were noted by the examiner, family, and patient (when able) and voltage was gradually increased in 0.1 v increments to a maximum of 2 v or when side effects were appreciated. It was noted that stimulation provided ipsilateral and contralateral effects as well as truncal effects that were difficult to localize. A 2 min wash-out period was utilized between contacts to mitigate confounding effects, as well as a 10 min wash-out period between electrodes. The patients’ PPN electrodes were then reprogrammed with a combination of beneficial contacts at the most clinically effective voltage in monopolar mode. GPi electrode stimulation was left unchanged during this visit.

Approximately 2 weeks following mapping of the PPN electrodes, the patient returned to clinic for mapping of the pallidal electrodes. Due to possible additive effects between PPN and GPi stimulation, the mapping of GPi was performed with the patient on their previous PPN stimulation settings. GPi mapping was performed unilaterally with each contact probed beginning at 0.2 v, 185 Hz, and 90 μs, with gradual increases by 0.1 v to a maximum of 4 v or when side effects were appreciated. Given the neuroanatomy and previous known response to circumferential stimulation in GPi (Gelineau-Morel et al., 2022), segmented contacts were not initially explored for mapping. It has been noted by the clinicians that while the effects of GPi stimulation are best seen longitudinally, initial effects on dystonia could be appreciated by the clinician, patient, and family. A wash out period of 5 min without stimulation was given between contacts and 30 min between electrodes. Effects of unilateral stimulation were predominantly on the contralateral side of the body. The patient was then reprogrammed with the most effective monopolar pallidal stimulation contacts at the most clinically effective frequency. As none of the three patients experienced significant worsening of dystonia interim to this appointment their PPN stimulation was left unchanged at this time.

#### 2.4.2. Programming

Following mapping visits, the patients were then seen every 1–4 weeks for further programming to optimize response. For the first 3 months, minimal changes were made to GPi except to gradually increase voltage as tolerated to therapeutic level of 3 v given longitudinal effects associated with pallidal stimulation. If significant side effects were appreciated, monopolar stimulation was often switched to bipolar or pulse width was decreased to limit current spread.

During the initial months of programming, focus was given to PPN. It was appreciated in all subjects that PPN stimulation was initially noted to have a transitory response on axial posturing as well as orofacial dyskinesias with any stimulation on beneficial contacts, but then with re-emergence of these concerns after several days. Patients 1 and 2 both experienced transient significant improvement in axial posturing for 48–72 h after programming visits with subsequent worsening of posturing to levels seen prior to surgical intervention following several programming visits. Patient 1 experienced an episode of worsening axial dystonia greater than levels seen preoperatively approximately 8 months after implantation, though it was unclear if this was related to worsening of his underlying progressive disease, as reprogramming following this occurrence provided substantial sustained benefit. These transient benefits with return to baseline necessitated frequent reprogramming, initially in clinic, but subsequently changes in parameters and stimulation contacts utilizing different groups through the patient programmer. If side effects were appreciated including orofacial dyskinesias or parasthesias, alternative contacts were trialed as well as consideration was made for bipolar stimulation to limit spread. Due to manufacturer limitations preventing utilization of bipolar stimulation on segmented contacts this was rarely utilized and instead alternative segmented contacts were trialed, as well as only constant voltage programming, as constant current programming is not currently allowed utilizing the combination of Sensight electrodes and the Activa RC generator. Cycling parameters were also trialed to limit neural plasticity (MacLean and Sanger, 2023), given concerns of acclimation, with patients noting to have different responses to different cycles, and requiring frequent adjustments and on/off cycling to achieve longer benefit as noted in their postoperative scores.

Given the wide range of frequencies reported in PPN stimulation (Ricciardi et al., 2019), once optimal contacts were identified stimulation was trialed at low (10–60 Hz), mid-range (60–95 Hz), and high (180–250 Hz) frequency. Patient 1 responded best to high frequency stimulation (200–250 Hz), while patients 2 and 3 responded best to low frequency stimulation (30–50 Hz.) All patients were noted to require only low voltage (< 1 V) stimulation in PPN for benefit, including up to 12 months post-implantation (Table 2).

## 3. Results

### 3.1. Benefits

**Figure 2:**
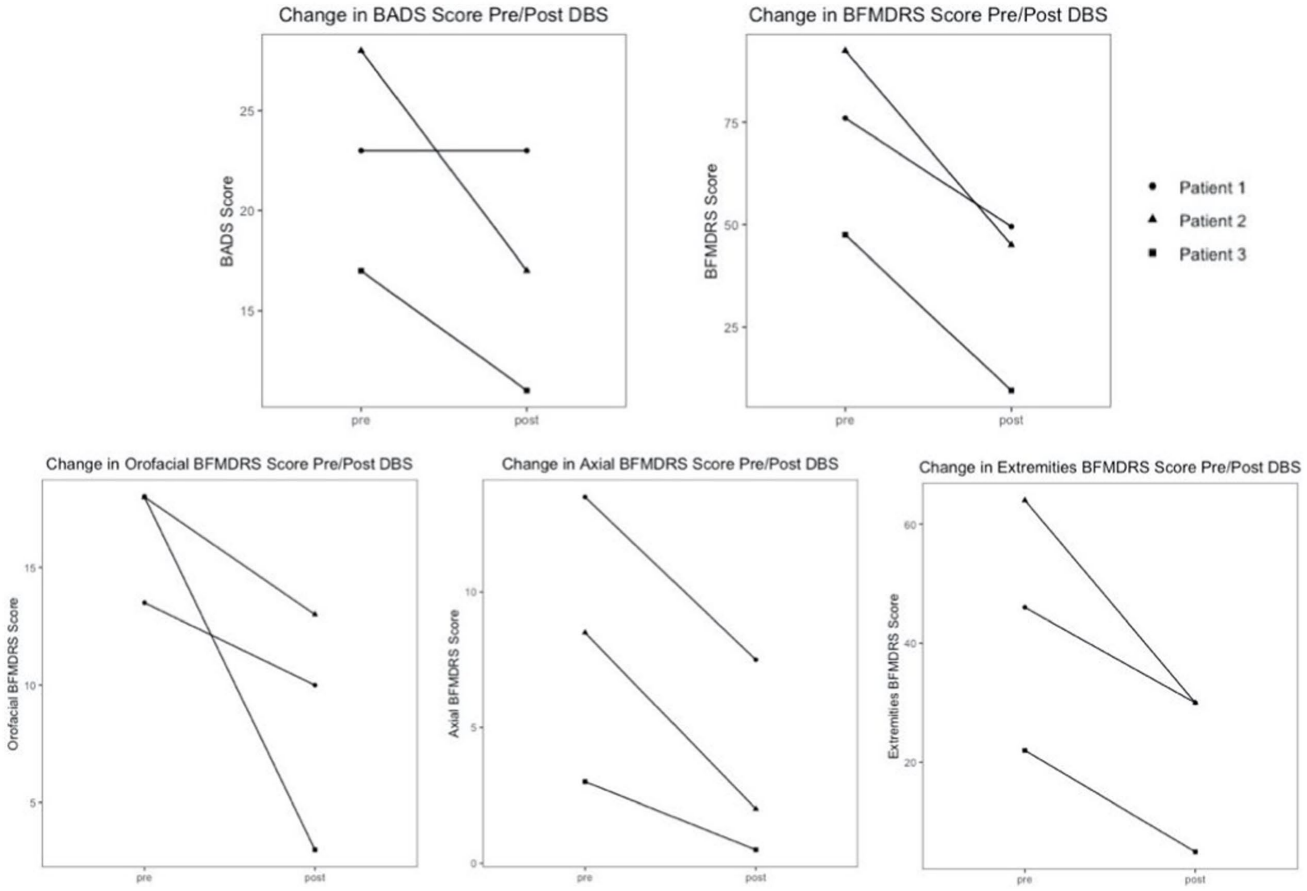
BADS and BFMDRS global/component motor scores pre- and post-operatively with combined PPN/GPi stimulation.

Patients were assessed between 3 and 12 months postoperatively after having been stable on programming for at least 2 weeks to mitigate transient effects as reported. All subjects demonstrated significant improvement in the BFMDRS Motor score. Specifically, Patient 1 showed a 34.9% decrease, Patient 2 showed a 51.4% decrease, and Patient 3 showed an 80.0% decrease. Patients 2 and 3 also demonstrated improvement in the BADS, 39.3 and 35.3%, respectively. Patient 1 did not show any change in BADS between the pre- and post-operative time points. Average improvement on the BFMDRS was 55.4%, and average improvement on BADS was 24.9%. It is noted there is a wide range in the degree of improvement likely related to the heterogeneity of the patients’ underlying conditions and symptoms. The lack of change seen in the BADS in patient 1 is likely related to the underlying severity of his dystonia and overflow component, which is not assessed in this scale, as well as its limitations in pediatric patients with other cooccurring tone concerns (Heringer et al., 2010). The response of all patients is shown in Table 3, as well as in Figure 2.

**Table 3:** Clinical response to combined PPN and GPi stimulation.

	BFMDRS motor score		BADS	
	Pre-surgical	Post-surgical	Pre-surgical	Post-surgical
Patient 1	76	49.5	23	23
Patient 2	92.5	45	28	17
Patient 3	47.5	9.5	17	11

Additionally, component scores of the BFMDRS were separated into three categories to assess response in subgroups of relevant dystonic features: orofacial, axial, and extremities. The orofacial component is the sum of the scores for mouth, speech, and swallowing. The axial component is the sum of the scores for neck and trunk regions. The extremities component is the sum of scores for bilateral arms and legs. All patients demonstrated improvement in orofacial, axial, and extremities component scores. Patient 1 demonstrated 25.9% orofacial improvement, 44.4% axial improvement, and 34.8% improvement in extremities. Patient 2 demonstrated 27.8% orofacial improvement, 76.5% axial improvement, and 53.1% improvement in extremities. Patient 3 demonstrated 83.3% orofacial improvement, 83.3% axial improvement, and a 77.3% improvement in extremities. The average response in each category across all subjects was: 45.7% orofacial improvement, 68.1% axial improvement, and 55.1% improvement in extremities. Patients 2 and 3 experienced significant improvement in oral-pharyngeal dystonia evident on their scale scores. Patient 3 has experienced resolution of previous concerns for choking, as well as significant improvements in speech. Component scores for all patients are shown in Table 4, as well as in Figure 2.

**Table 4:** Assessment of orofacial and axial response to combined PPN/GPi stimulation.

	Orofacial		Axial		Extremities	
	Pre-surgical	Post-surgical	Pre-surgical	Post-surgical	Pre-surgical	Post-surgical
Patient 1	13.5	10	13.5	7.5	46	30
Patient 2	18	13	8.5	2	64	30
Patient 3	18	3	3	0.5	22	5

Patients and parents of patients were also asked if they thought deep brain stimulation improved their (or their child’s) quality of life utilizing a Likert scale of “improved,” “no change,” or “worsening” quality of life concurrent with objective video scoring by the BADS and BFMDRS. Subjective quality of life improvements were noted by patient 1 and patient 3. Patient 2 could not answer due to cognitive/communicative limitations. The families of all three patients noted improvement in quality of life following DBS, with patient 2 family’s particularly noting an improvement in sleep patterns including decreased wake after sleep onset.

### 3.2. Side effects

Similar to the effects commonly reported with DBS programming of various targets (Zarzycki and Domitrz, 2020), patients experienced parasthesias and worsening dystonic posturing with initial mapping and probing of the PPN stimulation contacts. In possible relation to the urge urinary incontinence previously reported in the Parkinson’s disease cohort (Thevathasan et al., 2018), subject 2 experienced urinary retention of greater than 8 h during wakefulness with stimulation of specific contacts in PPN. This was replicated twice including with blinding of the patient and family utilizing previously set groups. Similar to the adult literature, we hypothesize this is likely related to the nearby pontine micturition center (Aviles-Olmos et al., 2011). Due to the cognitive limitations of the patient, it is impossible to characterize if retention is related to lack of urge.

None of the subjects experienced any perioperative or postoperative complications.

## 4. Discussion

Although pallidal DBS is well-established as the recommended target for Parkinson’s disease and DYT1 dystonia, its utilization and targeting for other conditions has been mixed (Andrews et al., 2010). Variation in underlying etiology and clinical presentation of dystonia, particularly in many of the pediatric-onset dystonia conditions, further complicates the issue and increases the likelihood that a singular target is insufficient. Additionally, target choice is especially important depending on the physical distribution of dystonia. Pallidal and thalamic targets have shown promise in alleviating motor components of dystonia related to limbs and ambulation but have displayed inferior and often incomplete response to orofacial and axial presentations (Castelnau et al., 2005). This incomplete response has necessitated the exploration of alternative DBS targets in treatment of orofacial and axial dystonia.

Based on previous trials of PPN stimulation in PD patients (Thevathasan et al., 2018), we trialed PPN as a DBS target in three patients with childhood-onset dystonia. Despite the varying nature of etiologies underlying the three subjects presented in this report, commonalities in symptomatology included strong axial and orofacial dystonic components, motivating the choice to use PPN as an exploratory target in conjunction with pallidal DBS. All three subjects showed marked improvements with combined pallidal and PPN stimulation, captured by the BADS and BFMDRS Motor Score. Orofacial, axial, and extremities BFMDRS component scores improved for all three patients. The improvement in both total and extremities component scores suggests that the predicted beneficial effects of pallidal DBS on dystonia in the extremities is preserved with combined PPN and GPi DBS. Additional subjective reports of improved quality of life and sleep present avenues for further examination including utilizing overnight polysomnography to further evaluate sleep changes as have been performed in the adult literature.

Figure 2 demonstrates that the range of percent improvements in motor scores across the cohort pre and post DBS is extremely wide, 45.1% difference between patient 1 and 3 in the BFMDRS, and 39.3% difference between patients 1 and 2 in the BADS scale. This highlights the amount of variation that is present in secondary dystonia, even when there are similarities in symptomatology, and emphasizes the importance of individualized programming post-operatively.

There are no reported cases of DBS in *MEPAN* hence we are unable to compare the patient’s response to PPN to traditional stimulation within his condition. Limited literature in the GA1, pediatric population has shown mixed response to pallidal deep brain stimulation with a range of change in the BADS score of 0–18% (Shlobin et al., 2023), significantly lower than the 39.3% noted in patient 2 with combined PPN and Gpi DBS.

Of the three patients investigated in the report, maximal response was achieved in the third patient. Patient 3 was diagnosed with atypical PKAN, and achieved an 80.0% improvement in BFMDRS motor score, with 83.3% improvements in both orofacial and axial components, and 77.3% improvement in extremities. PKAN is known to respond to pallidal stimulation, as evidenced by a previous study of six subjects with both classic and atypical PKAN receiving bilateral pallidal stimulation. The study reported an average 65.1% improvement on the BFMDRS motor score in four subjects with classic PKAN, and average 85.0% improvement on the BFMDRS motor score in two subjects with atypical PKAN (Castelnau et al., 2005). BFMDRS component scores were not reported, although suboptimal benefit in speech was noted. The improvement in global motor score with combined stimulation shown in this report is comparable to improvements seen with pallidal stimulation alone in PKAN. The 5% difference in benefit achieved in reported atypical PKAN scores could be explained by the difference in postoperative time point used, as scores reported for patient 3 in this study were measured 3 months postoperatively while scores reported in literature were measured at a minimum of 6 months postoperatively. Since the response to GPi stimulation is best observed longitudinally, further evaluation of combined PPN and GPi stimulation in the long-term is indicated. However, the degree of response in orofacial and axial areas shown in patient 3 highlights the positive clinical effects conferred by the addition of PPN stimulation.

The mechanisms of action of DBS on dystonia are not currently well understood (Lozano et al., 2019). GPi is considered to be the major output nucleus of the basal ganglia, exerting influence on both the thalamocortical loop via ventrolateral thalamus, and the brain stem—spinal cord via connections to PPN. In dystonia, there is evidence of signal abnormalities in the pallidum, suggesting that a possible mechanism of GPi DBS is that it alters or overrides these pathological signals without restoring normal function (Ostrem and Starr, 2008). Various human and non-human primate studies suggest that there is pathological underactivity of the PPN in both PD and dystonia, possibly related to cholinergic neural loss or overactivity of GABAergic projections from the GPi (Nowacki et al., 2019; Thevathasan and Moro, 2019; Su et al., 2022). A possible explanation for the motor benefits yielded by PPN DBS is that it partially ameliorates this depressed activity. Additionally, a key feature of development of dystonia is the imbalance between striatal dopamine and acetylcholine systems (Su et al., 2022). The PPN has extensive projections to dopaminergic neurons in the substantia nigra pars compacta, which could further explain the effect of PPN on motor function (Nowacki et al., 2019), as well as the presence of acetylcholine releasing neurons within PPN (Rye, 1997). The combined stimulation of GPi and PPN could play a role in stabilizing GABAergic, dopaminergic, and cholinergic interactions between basal ganglia, striatal, and PPN neurons. Although the exact mechanism of DBS, and the mechanisms of combined DBS, are unknown, it is possible that combined pallidal and PPN stimulation provide a coactivation effect that improves DBS outcomes for axial and orofacial symptoms. Further understanding of the mechanisms of DBS in single areas, as well as the interplay between DBS of multiple targets, could be very useful in establishing a methodology for optimal programming of PPN DBS, especially considering the transient effects of PPN DBS and variation in effective stimulation frequency reported in this cohort.

This cohort series is limited by its small and heterogenous patient population. Additionally, due to utilization of double bilateral stimulation in the subjects as part of typical clinical programming, we cannot adequately assess the results of PPN stimulation alone vs. in conjunction with pallidal stimulation. This assessment was particularly limited in clinical setting as subjects did not tolerate PPN stimulation being turned off, including when blinded to this change, with immediate worsening of axial dystonia. We also cannot ascertain if unilateral stimulation alone would have been sufficient for the clinical improvement appreciated by the patients and their families. Despite these limitations, this report describes the first known cases of DBS targeting of PPN in pediatric patients. While programming of PPN in combination with pallidal stimulation is complex and challenging, it may provide additional benefit in a subset of patient with axial and orofacial symptoms. Despite the difficulty associated with targeting PPN using standard techniques, all patients tolerated the procedure well, and no perioperative complications with DBS placement are reported. Patients displayed some sensitivity to stimulation frequencies and voltages, indicating that programming plays a strong role in success of the PPN target. However, all patients showed clinically significant improvements in BFMDRS scoring post-operatively, especially in scale subcategories associated with axial and orofacial features of dystonia. This suggests that PPN is a safe DBS target for pediatric secondary dystonia, and that co-stimulation of GPi and PPN may be an effective treatment paradigm for some components of dystonia that are insufficiently treated with GPi stimulation alone.

## Data availability statement

The data presented in this study are available on request from the corresponding author, subject to patient consent to privacy. The data are not publicly available due to patient privacy.

## Ethics statement

The studies involving humans were approved by Children’s Hospital of Orange County Human Subjects Institutional Review Board Approval 200330, 13 July 2020 to 24 July 2024. The studies were conducted in accordance with the local legislation and institutional requirements. Written informed consent for participation in this study was provided by the participants’ legal guardians/next of kin. HIPAA authorization for use of protected health information was obtained.

## Author contributions

JM: Conceptualization, Formal analysis, Methodology, Validation, Writing – original draft, Writing – review & editing. JN: Data curation, Formal analysis, Investigation, Methodology, Writing – original draft, Writing – review & editing. JD: Writing – review & editing, Conceptualization. AZ: Writing – review & editing, Writing – original draft. JK: Conceptualization, Writing – review & editing, Methodology. ML: Conceptualization, Methodology, Writing – review & editing. JO: Conceptualization, Methodology, Writing – review & editing. TS: Conceptualization, Formal analysis, Methodology, Writing – review & editing.
